# Work environment and quality of nursing care in primary health care: a scoping review

**DOI:** 10.1080/07853890.2021.1896072

**Published:** 2021-09-28

**Authors:** Maria Adelaide Martins, Pedro Bernardes Lucas

**Affiliations:** a Agrupamento de Centros de Saúde da Amadora, Amadora, Portugal; b Unidade de Investigação e Desenvolvimento em Enfermagem da Escola Superior de Enfermagem de Lisboa, Lisboa, Portugal

## Abstract

**Introduction:**

The nursing practice environment (NPE) has been analysed and is now recognised as a variable that influences the results of nursing care, since the promotion of favourable environments is fundamental to the quality of care optimisation [1]. The development and implementation of a positive NPE in primary health care (PHC) improve nurses’ well-being, influence nursing satisfaction, reduce nurses’ intention to leave, improve nursing care outcomes and patient care quality [2]. Investigating the characteristics of the NPE and the quality of care in PHC has become a priority. The aim was to map and analyse the scientific evidence on the NPE and the quality of nursing care (QNC) in PHC.

**Materials and Methods:**

We conducted a scoping review, according to Joanna Briggs Institute’s approach [3]. A three‐step search was carried out: (a) keywords search within MEDLINE and CINAHL; (b) keywords search within COCHRANE, PSYCOLOGY AND BEHAVIOUR SCIENCES, and COLLECTION B-ON; (c) literature search of references lists to identify additional studies. From published literature in English and Portuguese, with no date restrictions, we selected articles considered eligible to study the NPE and QNC in the context of PHC. A qualitative content analysis was performed.

**Results:**

We retrieved 289 records and selected 12 papers, including quantitative and qualitative studies. Our analysis revealed that positive NPE improves nurses’ satisfaction and, consequently, improves QNC. Additionally, some characteristics of the NPE should be improved, such as the participation of nurses in management decisions, leadership, education, guidance, support and recognition of the work developed by nurses. Autonomy, control over the environment and collaboration between health professionals and managers improve teamwork and QNC.

**Discussion and Conclusions:**

A positive NPE culture in PHC is highlighted as one of the key factors for the satisfaction, retention and recruitment of nurses and is associated with the improvement of the QNC. Concurrently, the development of a teaching-learning culture between managers and the nursing team develops and improves professional skills, with the consequent improvement of nursing practices.
